# Prevalence and occupational risk of hip osteoarthritis and rotator cuff lesions – claims data analysis

**DOI:** 10.13075/ijomeh.1896.02663

**Published:** 2026

**Authors:** Kristina Hagenström, Katharina Müller, Theresa Klinger, Matthias Augustin, Albert Nienhaus

**Affiliations:** 1 German Social Accident Insurance Institution for the Health and Welfare Services (BGW), Hamburg, Germany; 2 University Medical Center Hamburg-Eppendorf (UKE), Institute for Health Services Research in Dermatology and Nursing (IVDP), Hamburg, Germany

**Keywords:** osteoarthritis, epidemiology, occupational risk, statutory health insurance, work exposure, RCL

## Abstract

**Objectives:**

Hip osteoarthritis (OA) and rotator cuff lesions (RCL) are major musculoskeletal disorders that cause chronic pain, reduced mobility, and work incapacity. While certain occupational groups have been studied, limited data exist on other physically demanding professions typically performed by women, such as healthcare, childcare, and hairdressing. This study examines the prevalence and occupational risks of hip OA and RCL in Germany.

**Material and Methods:**

A retrospective analysis was conducted using anonymized, large-scale, nationwide claims data (2016–2020). Individuals aged 18–65 years diagnosed with OA (International Classification of Diseases and Related Health Problems [ICD-10]: M16) or RCL (ICD-10: M75) were identified. Physically demanding occupational groups were classified according to the German Federal Employment Agency and compared to a propensity score-matched control group of office workers.

**Results:**

Between 2016 and 2020, the prevalence of hip OA increased from 1.8% to 1.9%, and RCL from 4.4% to 4.6%. Higher prevalence rates for RCL were found in exposed occupations (4.9%). Increased risks were observed in elderly care (OA: odds ratio[OR] = 1.33, RCL: OR = 1.49) and in health and nursing care (OA: OR = 1.33, RCL: OR = 1.27) compared to office workers with lower physical exposure. Restricting the analysis to women yielded similar effect estimates.

**Conclusions:**

The findings highlight an elevated occupational risk for hip OA and RCL in physically demanding professions such as nursing. Targeted prevention, ergonomic interventions, and early screening are essential to reducing work disability and improving long-term health outcomes.

## Highlights

- Elderly and nursing care workers face significantly increased musculoskeletal risks.
- Physically demanding jobs show higher odds for hip osteoarthritis (OA) and rotator cuff lesions (RCL).
- Risk patterns persist among women in female-dominated professions.
- Claims data reveal rising prevalence of hip OA and RCL in German workforce.

## INTRODUCTION

Hip osteoarthritis (OA) and rotator cuff lesions (RCL) pose significant health challenges, with considerable individual and societal costs. These conditions impact both the general population and specific occupational groups exposed to increased risk due to their working conditions [1, 2]. Their effects range from chronic pain and limited mobility to a significant reduction in quality of life and the inability to work.

The prevalence of hip OA is estimated at 2.4–8.6% [2–6]. Furthermore, prevalence is higher in women and increases with age [4, 6, 7]. Regional differences have been demonstrated worldwide, with the highest rates recorded in Europe [5, 8]. The prevalence of RCL is estimated 20.7–22.2% [9, 10]. It also increases with age [11–14] and is more prevalent in women [15–17]. However, the studies are only comparable to a limited extend due to the differences in study design and populations.

Previous studies have shown that certain occupational groups are predisposed to developing musculoskeletal diseases due to specific working conditions [18]. Since 2021, hip OA and RCL have been recognized as occupational diseases [19, 20]. However, the scientific justification for this recognition primarily focused on occupational groups with prolonged high exposure, including agriculture and forestry, animal husbandry and horticulture, construction, and the food, textile, metal, and electrical industries. Other physically demanding professions, such as healthcare and nursing, hairdressing, and physiotherapy, have not yet been adequately studied.

However, the literature highlights the need for further research into these conditions and their occupational risk factors. In this context, it is particularly important to examine the prevalence, risk factors, and consequences of these conditions in exposed occupational groups to develop targeted prevention and intervention strategies. The aim of this study is to analyze the epidemiological situation of hip OA and RCL in both the general population and in occupational groups that have been understudied but are likely to have high exposure in Germany, using a large, nationwide claims database.

## MATERIAL AND METHODS

### Data source

This is a retrospective secondary data analysis based on an anonymized random 40% sample from a large, nationwide statutory health insurance (SHI) provider, DAK-Gesundheit (DAK-G) (N = 2 885 984, 56.8% women, mean (M) age 48.6 years). The whole sample was reduced to a 40% sample by the insurance in compliance with the principle of social data minimization. The dataset includes all insured persons who were covered for at least 1 day in January 1, 2016 – December 31, 2020. This sample includes all insured individuals, regardless of their employment status or age. This includes employed and unemployed persons, retirees, and co-insured family-members.

### Study population

The study population consists of insured individuals aged 18–65 years who were insured for ≥1 day/quarter of the observation year. Individuals with family insurance and pensioners were excluded. Insured persons who died during the observation period were not excluded from the analyses.

The study included individuals diagnosed with OA (International Classification of Diseases and Related Health Problems [ICD-10-GM: M16] or RCL [ICD-10-GM: M75]) who had received ≥1 confirmed outpatient or inpatient diagnosis within the observation year, whether as a primary or secondary diagnosis.

Occupation was classified according to the most recent classification of occupations issued by the German Federal Employment Agency [21]. The following occupational groups were analyzed: healthcare and nursing, midwifery and maternity care, elderly care, emergency services, hairdressers, physiotherapy (including masseurs), childcare, and dental assistants (Table 1). For comparison, office workers (e.g., in natural sciences, geography, IT, business organization, accounting, law, and administration) were selected as a control group due to their lower physical exposure and comparable qualifications. To ensure comparability in qualifications, the authors restricted the analysis to occupations with a fifth-digit. This fifth digit denotes the skill level of an occupation, with levels 1–3 representing helper, trained, and technically skilled positions.

**Table 1.** Relevant classification codes of the occupational and control groups issued by the German Federal Employment Agency [21] used in the retrospective secondary data analysis on insured individuals aged 18–65 years diagnosed with hip osteoarthritis and rotator cuff lesions (N = 2 885 984)

Occupational group and code number	Occupational title	Hip osteoarthritis	Rotator cuff lesion	Control group
Health and nursing care				
8130	professions in healthcare and nursing (without specialization)	X	X	
8131	professions in specialized nursing	X	X	
8132	professions in specialized pediatric nursing			
8138	healthcare and nursing professions (other specific job specification)	X	X	
Obstetrics and maternity care				
8135	professions in obstetrics and maternity care	X	X	
Elderly care				
8210	professions in geriatric nursing (without specialization)	X	X	
8218	professions in geriatric care (other specific job specification)	X	X	
Emergency services				
8134	occupations in emergency services	X	X	
Hairdressers				
8231	hairdressing professions		X	
Physiotherapy including masseurs				
8171	occupations in physiotherapy	X	X	
Childcare				
8311	occupations in childcare and education	X	X	
Dental assistants				
81112	dental assistants – specialized activities		X	
81113	dental assistants – complex specialized activities		X	
Natural sciences, geography and information technology				
41	math, biology, chemistry and physics professions			X
42	geology, geography and environmental protection professions			X
43	computer science, information and communication technology professions			X
Business organisation, accounting, law and administration				
71	business management and organization occupations			X
72	financial services, accounting, and tax consulting occupations			X
73	legal and administrative occupations			X

X indicates that the respective code was allocated to the hip group, rotator cuff group, or control group, and was therefore included in the relevant endpoint and comparison analyses.

The present study involved the comprehensive documentation of all inpatient stays of prevalent persons with the respective target disease in the respective observation years. Furthermore, an analysis was conducted to ascertain the impact of the respective illness on the capacity of individuals to engage in work activities. The number of stays or periods of incapacity for work, and the respective duration in days, were analyzed.

The study was conducted according to national guidelines for the use of administrative databases [22, 23]. According to those guidelines, no approval of an ethical committee is required.

### Statistical analysis

The administrative annual prevalence rates were estimated as percentages with 95% confidence intervals (CI) for the observation years 2016–2020. Estimates were standardized by age and sex according to the German Federal Statistical Office (Destatis) as of December 31 of each respective year. Baseline characteristics (age, sex, settlement structure, and relevant comorbidities) were presented as percentages for categorical variables. The employment of statistical tests, including the χ² test, the t-test and the Wilcoxon rank sum test, was utilized for the purpose of comparison of the occupational cohort with all insured persons diagnosed with the target diseases.

In order to adjust for differences in baseline characteristics between physically exposed and non-exposed occupations, propensity score (PS) matching was employed [24]. A 1:1 nearest-neighbor matching approach (greedy matching) was used, controlling for sex, age, settlement structure (rural, semi-rural, urban), and predefined comorbidities [25–31] (Table 2). Missing values in the settlement structure variable were treated as a separate category. To ensure the validity of the PS matching, several consistency checks were conducted, including assessments of potential confounding and the positivity assumption (PA) (i.e., ensuring that each participant had a non-zero probability of being assigned to either exposure group). Consistency was ensured through pseudo-randomization, and confounding was minimized by excluding test subjects with extreme PSs. The distribution of PS values was visually verified using box plots. In order to analyze the relationship between occupational exposure and target disease, a logistic regression was performed and odds ratios (ORs) with corresponding 95% CI were calculated. All analyses were conducted using SAS v. 9.4 German (SAS Institute, Cary, NC, USA).

**Table 2.** International Classification of Diseases v. 10, German Modification (ICD-10-GM) diagnoses of pre-defined comorbidity used in the retrospective secondary data analysis on insured individuals aged 18–65 years diagnosed with hip osteoarthritis and rotator cuff lesions (N = 2 885 984)

ICD-10-GM	Name	Coxarthrosis	Rotator cuff lesions
E66	obesity	X	X
Q65	congenital deformities of the hip (hip displasia)	X	
Inflammatory rheumatic diseases			
G72.4	inflammatory myopathies (idiopathic inflammatory myopathies)	X	X
L40.5, M07.0–M07.3, M09.0	psoriatic arthritis	X	X
M05	seropositive chronic polyarthritis (rheumatoid arthritis)	X	X
M06	other chronic polyarthritis	X	X
M31.3, M31.7	granulomatosis with polyangiitis, microscopic polyangiitis (ANCA-associated vasculitides)	X	X
M31.5	giant cell arteritis in polymyalgia rheumatica	X	X
M31.6	other giant cell arteritis	X	X
M33.0	juvenile dermatomyositis	X	X
M33.1	other dermatomyositis	X	X
M33.2	polymyositis	X	X
M34	systemic sclerosis (scleroderma)	X	X
M35.0	Sicca syndrome (Sjögren's syndrome)	X	X
M35.3	polymyalgia rheumatica	X	X
M45	ankylosing spondylitis (Bechterew's disease)	X	X
M60.8	other myositis (idiopathic inflammatory)	X	X
M60.9	myositis, unspecified (idiopathic inflammatory myopathies)	X	X
Other identified risk factors			
F32–F33	depression	X	X
F10	mental and behavioural disorders due to alcohol	X	X
F17	mental and behavioural disorders caused by tobacco	X	X

ANCA – antineutrophil cytoplasmic antibody.

X indicates that the respective code was allocated to the hip group, rotator cuff group, or control group, and was therefore included in the relevant endpoint and comparison analyses.

## RESULTS

### Study population

A total of 85.2% of the individuals in the relevant occupational groups were women. The proportion of women was particularly high in the occupational groups of obstetrics and maternity care, pediatric nursing, and dental assistants, with >90% of individuals in these groups being women (with the exception of emergency services, where men comprise 73.2%). The age of the individuals was M = 42.5 years with median (Me) 44 years and standard deviation (SD) 12.8 years. Due to the limited number of cases per disease, it was not possible to conduct further analyses for specific occupational groups, such as hairdressers, midwives, maternity nurses, pediatric nurses, and paramedics, for OA; and maternity nurses and pediatric nurses for RCL.

### Prevalence of hip osteoarthritis and rotator cuff lesions of the shoulder within the study population and the professional group

**Figure 1.**
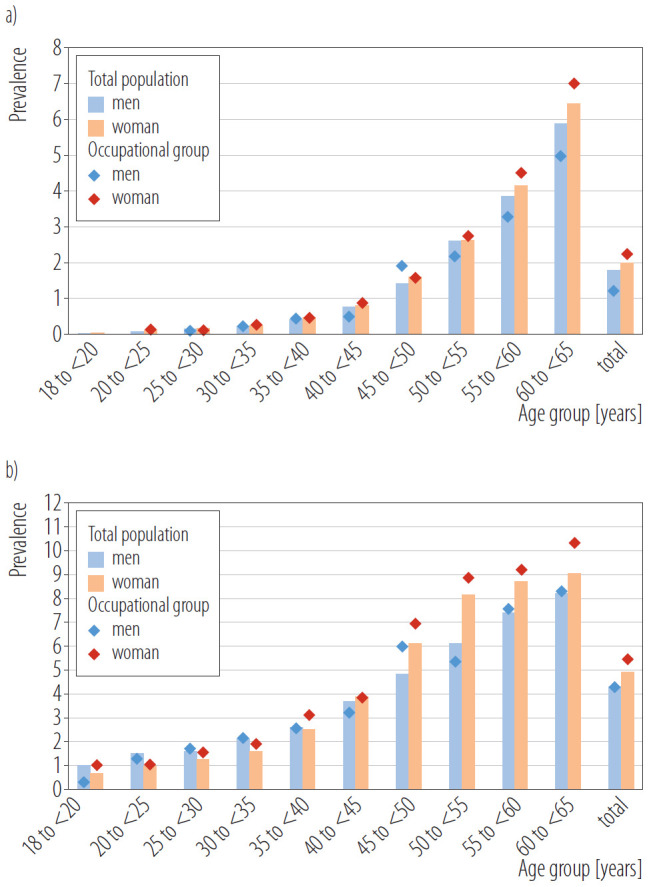
Standardized annual prevalence rate in percent of a) hip osteoarthritis (OA) and b) rotator cuff lesions (RCL) in the total population and occupational group by age and sex in 2020 in the retrospective secondary data analysis on insured individuals aged 18–65 years diagnosed with hip osteoarthritis and rotator cuff lesions (N = 2 885 984)

The standardized prevalence of OA increased slightly between 2016 and 2020, from 1.8% to 1.9%. This equates to around 970 000 adults aged 18–65 years in Germany in 2020. Similarly, RCL increased from 4.4% to 4.6%, over the same period, affecting around 2.35 million adults in this age group in Germany. The prevalence rates for RCL were slightly higher in the occupational groups analyzed, with 4.9%. In the general population women were more likely to suffer from both conditions than men (OA: 2.0% women vs. 1.8% men, RCL: 4.9% women vs. 4.3% men). The sex difference was particularly pronounced within the occupational group (OA: 2.1% women vs. 1.6% men, RCL: 5.4% women vs. 4.3% men) (Figure 1). Additionally, there was a continuous increase in prevalence with age, which increased the likelihood of developing each target disease. There was no significant difference in the mean age of individuals with and without the occupational group for both target diseases (OA: 55.4 years occupational group vs. 55.3 years total population, p = 0.971, RCL: 55.8% occupational group vs. 50.7% total population, p = 0.946). However, no age- or sex-related differences were found between the years, and the annual study populations' demographic composition remained stable throughout the observation period.

### Association between occupational group and hip osteoarthritis/rotator cuff lesions compared with a matched control group

**Figure 2.**
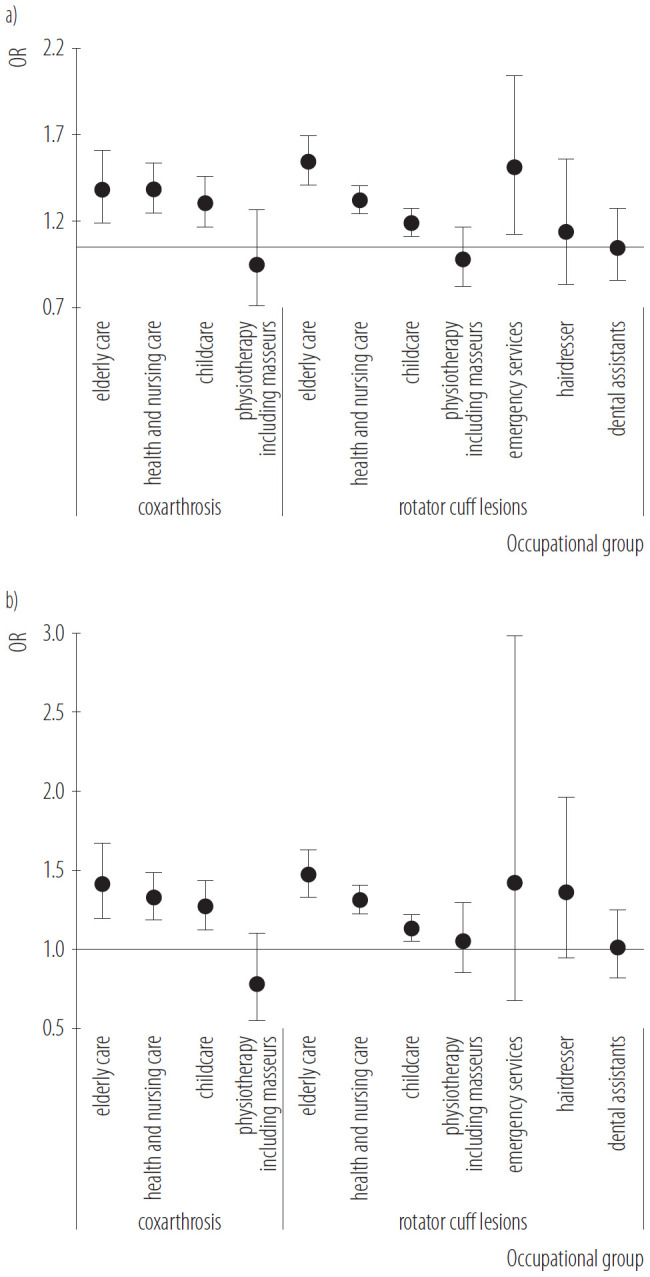
Risk of hip osteoarthritis and rotator cuff lesions in a) occupational group and b) occupational group in women compared to the control group (1:1 greedy-propensity score matching) in 2020 in the retrospective secondary data analysis on insured individuals aged 18–65 years diagnosed with hip osteoarthritis and rotator cuff lesions (N = 2 885 984)

The results showed that the odds of OA were increased by a factor of 1.33 (95% CI: 1.14–1.56) in the elderly care group compared with the control group. For RCL, the OR was 1.49 (95% CI: 1.36–1.64) in the elderly care group compared with the control group. The OR of OA for the healthcare and nursing occupational group was 1.33 (95% CI: 1.20–1.48), while the OR for the childcare occupational group was 1.25 (95% CI: 1.11–1.41). In these occupational groups, the odds of RCL were slightly lower (healthcare and nursing: OR = 1.27, 95% CI: 1.19–1.36; childcare: OR = 1.14, 95% CI: 1.06–1.22). When the data were analyzed for women only, there were only minor differences (except for hairdressers and emergency services) (Figure 2).

### Inpatient care and incapacity to work of hip osteoarthritis and rotator cuff lesions within the study population and the professional group

In the general population, hospitalization (OA: 0.1%, M±SD 8.5±3.2 days, RCL: 0.1%, M±SD 3.4±1.7 days) and incapacity to work (OA: 0.2% of cases, M±SD 98±142 days, RCL: 0.6% of cases, M±SD 48±90 days) are rare among individuals with these conditions. The proportion of people with RCL who were off work was slightly higher in occupations such as elderly care (1.1%) or health and social work (0.9%).

## DISCUSSION

With this current large-scale claims data analysis robust epidemiological data on OA and RCL in the general population and in exposed occupational groups with a high proportion of female workers in Germany were obtained. Compared to the literature, the data show lower prevalence rates than those reported in previous studies. For OA, the prevalence in 2020 was 1.9%. Literature estimates a markedly higher prevalence, ranging from 2.4% to 8.6% [2–6,15]. The methodologically comparable study by Kajos et al. reported similar results, with a prevalence of 2.2% in the general population in 2018 [2]. The higher prevalence rates in previously published studies are not fully comparable due to differences in study design, the selection of older age groups, and, as both prior research and the present data indicate, the significant increase in prevalence with age.

At 4.6% in 2020, the estimated prevalence of RCL is lower than previously published results, which range from 20.7% to 22.2% [9, 10]. However, both of these studies were based on selected populations from a single city in Japan. Similar results were found in the systematic literature review by Littlewood et al., which reported prevalence rates ranging 0.5–7.4%. The most recent study included in this review found a prevalence of 2.8% in 2001 [32].

The age and sex distribution indicates that, within the occupational groups studied, a higher proportion of women than men are affected by the target diseases compared to the general population. This discrepancy can be attributed to the disproportionate representation of women in these specific occupational groupsand the fact that women already exhibit higher baseline prevalence rates in the general population. Overall, the available data confirm previous findings that the prevalence of these diseases increases significantly with age and that women are more affected than men [4, 6, 7, 11–14, 16, 17].

People working in physically demanding occupations such as childcare, nursing, healthcare, and elderly care were 25–33% more likely to develop OA and 14–49% more likely to develop RCL than those with less exposure. This increased risk was more pronounced among women working in elderly care (OR = 1.33) for developing OA and among women working in nursing and healthcare (OR = 1.49) for developing RCL. A published study investigating the risk of developing OA in nursing occupations found a 57% higher probability after >10 years of exposure (hazard ratio 1.57, 95% CI: 1.46–1.69) [33]. Another study found that female child care workers were 70% more likely of develop shoulder and upper arm disorders compared to women from other occupations in the general population (OR = 1.70, 95% CI: 1.12–2.52) [34]. The effect estimates observed in the present study are lower, albeit still within the range of those reported previously. These differences may reflect variations in exposure definitions, occupational classifications, and follow-up duration across studies. Additionally, differences in healthcare utilisation and coding practices, as well as variations in occupational structures and job profiles across similarly labelled occupations, may limit the transferability of prevalence estimates and effect sizes derived from claims data. Given the widespread prevalence of physically demanding work in many occupations, even modest relative risks, such as those observed here, could result in significant population-attributable burdens. These findings therefore support the existing body of evidence linking occupational physical workload to degenerative joint disorders, while also highlighting the need to consider methodological heterogeneity when comparing effect sizes across studies.

Men who work in emergency services were more likely to suffer from RCL. However, this could not be confirmed for women in this occupational group, due to the fact that this occupation is predominantly performed by men (82.7%). A similar gender effect was found for hairdressers. Although the association was not significant in either group, female hairdressers had a higher estimated risk of RCL than hairdressers overall. This may be due to the fact that the few men considered (10.0%) changed jobs more frequently and no longer worked in this exposed occupation, resulting in a shorter exposure duration. In addition to these structural differences in the workforce, several occupation-specific factors may help to explain the observed gender differences. These patterns may reflect:

– an occupational effect (i.e., differences in physical workload intensity or task allocation within the same job title),
– a sex-specific vulnerability to similar biomechanical loads.

For example, in emergency services, men may be more frequently assigned physically demanding tasks, while women may more often be engaged in roles that focus on coordination or communication. Furthermore, anthropometric differences, such as body height, upper-limb reach, and upper-body muscle mass, may influence the distribution of mechanical load during load handling. Furthermore, for example rescue equipment is often designed with male anthropometry in mind, which may alter the biomechanical demands placed on women. These mechanisms cannot be tested with the available claims data, as task-specific workload, cumulative exposure, and anthropometry are not captured. Further primary studies or data linkage with detailed exposure assessment are required to disentangle differences in occupational exposure from sex-specific susceptibility.

Hospitalization and sick leave due to the target diseases were rare. Only in the occupational groups of elderly care and health and nursing care, which had the highest risk of illness compared to the control group, were there slightly more people on sick leave.

### Strengths and limitations

The fundamental strength of this analysis lies in the extensive SHI dataset, which has considerable scientific value due to its population coverage. However, it should be noted that data and methodological limitations must be taken into account when interpreting the results [23]. For example, the population groups of the different health insurance funds could differ [35]. To minimize these differences, the prevalence rates have been standardized by age and sex. As the data are claims data not collected for research purposes, only illnesses and care provided to insured individuals who actually used the healthcare system can be represented. Therefore, the prevalence rates may be higher than those identified in these analyses. The analysis is also limited by the fact that matching factors such as clinical parameters, individual health behavior and socioeconomic status cannot be taken into account, as they are not recorded in the social health insurance data. It should also be noted that the present dataset only takes into account the most recent status of the occupation code. This means that the duration of employment, and thus the cumulative physical exposure, cannot be assessed with the available data. Individuals who have changed occupations over time may therefore be misclassified with respect to their true long-term exposure. Importantly, this misclassification is expected to occur independently of disease status in both groups (occupational group and control group), and therefore represents non-differential misclassification. This type of bias typically reduces the strength of observed associations, implying that the true effect estimates may be stronger than those reported in this study.

## CONCLUSIONS

Osteoarthritis and RCL are common conditions that are becoming more prevalent in the general population and are expected to increase in certain occupational groups, particularly those with high physical exposure. The authors' findings suggest that occupational groups with expected high physical exposure, such as nursingand related healthcare activities, are more likely to develop OA or RCL than occupational groups with low physical exposure. The observed statistically significant OR were in the range of 1.2–1.5. Therefore, the data support the assumption that OA and RCL are multifactorial work-related diseases in nurses and childcare workers. Further studies with a more detailed exposure assessment instead of using job titles as a surrogate for exposure are needed to investigate whether OA and RCL qualify for an occupational disease in nurses and childcare workers. These studies could include prospective cohort studies or case-control studies with detailed exposure assessment, as well as studies linking primary and secondary data.

In addition, the authors' data confirm the need for targeted strategies to prevent or delay the onset of these diseases. Occupational groups with an increased prevalence of musculoskeletal disorders should be considered in future ergonomic risk assessments and targeted workplace interventions. Furthermore, regularly assessing trends in musculoskeletal disorders among high-exposure occupational groups could help identify an increase in disease burden at an early stage, supporting the timely implementation of preventive measures.
